# BM-MSC-derived exosomes alleviate radiation-induced bone loss by restoring the function of recipient BM-MSCs and activating Wnt/β-catenin signaling

**DOI:** 10.1186/s13287-018-1121-9

**Published:** 2019-01-15

**Authors:** Rui Zuo, Minghan Liu, Yanqiu Wang, Jie Li, Wenkai Wang, Junlong Wu, Chao Sun, Bin Li, Ziwen Wang, Weiren Lan, Chao Zhang, Chunmeng Shi, Yue Zhou

**Affiliations:** 1 Department of Orthopedics, Xinqiao Hospital, Army Medical University (Third Military Medical University), Chongqing, 400038 People’s Republic of China; 2 Institute of Rocket Force Medicine, State Key Laboratory of Trauma, Burns and Combined Injury, Army Medical University(Third Military Medical University), Chongqing, 400038 People’s Republic of China

**Keywords:** Radiation, Exosomes, Bone marrow mesenchymal stem cell, Differentiation, β-Catenin

## Abstract

**Background:**

Radiotherapy to cancer patients is inevitably accompanied by normal tissue injury, and the bone is one of the most commonly damaged tissues. Damage to bone marrow mesenchymal stem cells (BM-MSCs) induced by radiation is thought to be a major cause of radiation-induced bone loss. Exosomes exhibit great therapeutic potential in the treatment of osteoporosis, but whether exosomes are involved in radiation-induced bone loss has not been thoroughly elucidated to date. The main purpose of this study is to investigate the role of exosomes derived from BM-MSCs in restoring recipient BM-MSC function and alleviating radiation-induced bone loss.

**Methods:**

BM-MSC-derived exosomes were intravenously injected to rats immediately after irradiation. After 28 days, the left tibiae were harvested for micro-CT and histomorphometric analysis. The effects of exosomes on antioxidant capacity, DNA damage repair, proliferation, and cell senescence of recipient BM-MSCs were determined. Osteogenic and adipogenic differentiation assays were used to detect the effects of exosomes on the differentiation potential of recipient BM-MSCs, and related genes were measured by qRT-PCR and Western blot analysis. β-Catenin expression was detected at histological and cytological levels.

**Results:**

BM-MSC-derived exosomes can attenuate radiation-induced bone loss in a rat model that is similar to mesenchymal stem cell transplantation. Exosome-treated BM-MSCs exhibit reduced oxidative stress, accelerated DNA damage repair, and reduced proliferation inhibition and cell senescence-associate protein expression compared with BM-MSCs that exclusively received irradiation. Following irradiation, exosomes promote β-catenin expression in BM-MSCs and restore the balance between adipogenic and osteogenic differentiation.

**Conclusions:**

Our findings indicate that BM-MSC-derived exosomes take effects by restoring the function of recipient BM-MSCs. Therefore, exosomes may represent a promising cell-free therapeutic approach for the treatment of radiation-induced bone loss.

**Electronic supplementary material:**

The online version of this article (10.1186/s13287-018-1121-9) contains supplementary material, which is available to authorized users.

## Background

Radiotherapy has become an essential treatment for cancer patients. However, the use of radiotherapy will cause injury to normal tissues while it affects cancerous tissues. The bone is the most commonly irradiated normal tissue, and radiotherapy increases the risk of osteoporosis and fracture in cancer patients [1–3]. Irradiation-induced fractures are common and clinically significant, especially in patients undergoing radiation therapy in the pelvic region, and pelvic fractures are an important cause of disability and death in the elderly [4, 5]. However, the mechanisms of radiation-induced bone loss are not completely understood.

Numerous studies have demonstrated that exposure to radiation leads to bone loss by affecting the survival and differentiation potential of bone marrow mesenchymal stem cells (BM-MSCs) [6–8]. Radiation causes DNA damage, chromosomal aberrations, increased reactive oxygen species, and cell senescence on BM-MSCs, hindering the capacity of BM-MSCs to proliferate [9–11]. In addition, following irradiation, BM-MSCs preferentially differentiate into adipocytes rather than osteoblasts, which ultimately leads to fat accumulation and bone loss [12, 13]. Given that BM-MSCs play an important role in maintaining bone homeostasis, altered proliferation and differentiation of BM-MSCs is one main cause of radiation-induced bone loss [14, 15]. Therefore, we seek to understand how to reduce the damage to BM-MSCs and restore their differentiation ability to alleviate radiation-induced bone loss.

Mesenchymal stem cell transplantation (MSCT) has been used to treat a variety of human diseases [16–18] and is an effective treatment for osteoporosis in an animal model [19–21]. Studies have demonstrated that MSCT ameliorates osteoporosis by recovering the function of recipient BM-MSCs. Although MSCT exhibits great potential in the treatment of various diseases, defects and risks still exist, such as storage difficulties, immune rejection, and carcinogenic risk [19, 22]. A recent study indicates that MSCT works via secretion of paracrine factors rather than direct homing to injured tissues [24]. Exosomes are specific secretory vesicles involved in the paracrine effects of MSCs, and studies have demonstrated that the exosomes play a repairing role to the same extent as MSCT [23, 25]. Compared with MSC transplantation, exosome therapy is a better choice given its improved safety, reduced immune rejection, and easier storage, delivery, and management [24].

Exosomes, a component of paracrine secretion, are vesicles with a bilayer membrane structure that is 40–100 nm in diameter that contain functional mRNA, microRNA, and proteins that exhibit cytoprotective effects to enhance tissue repair [26]. Various researchers have confirmed that MSC-derived exosomes exhibit protective activity and are effective in animal models of myocardial infarction [27], liver failure [28], and ischemic/reperfusion injury [29]. In addition, exosome transplantation has also proven to be effective in the treatment of bone loss-related diseases [30–32]. However, whether BM-MSC-derived exosomes could reduce radiation-induced bone loss remains unknown.

In this study, we demonstrate that MSCT and BM-MSC-derived exosome transplantation could rescue bone loss of rats following irradiation, and exosomes can alleviate radiation-induced damage to BM-MSCs. We also found that exosomes could restore the balance of adipogenesis and osteogenesis of irradiated BM-MSCs by activating the Wnt/β-catenin pathway.

## Materials and methods

### Animals

All animal studies were conducted in accordance with the ethical standards set by the Declaration of Helsinki and approved by the Laboratory Animal Welfare and Ethics Committee Of the Third Military Medical University. Three-month-old female Sprague-Dawley rats (Tengxin Biotechnology Co. Ltd., Chongqing, China) were randomized into five groups (*n* = 6): day 0 group, rats with no treatment and sacrificed on day 0; day 28 group, rats with no treatment and sacrificed on day 28; R day 28 group, rats received 16 Gy irradiation on day 0 and sacrificed on day 28; R+MSCT day 28 group, rats received 16 Gy irradiation and MSC transplantation and sacrificed on day 28; and R+Ex day 28 group, rats received 16 Gy irradiation and exosome transplantation and sacrificed on day 28. Rats received a total of 16 Gy radiation on day 0 to the knee joint region of the left hind limb using Co-60 (Additional file 1: Figure S1). The radiation rate was 0.56 Gy/min (Radiation Center, Army Medical University). After irradiation, the R+MSCT group immediately received an MSC transplantation (1 × 10^6^ cells), and the R+Ex group received an exosome transplantation (1.6 mg/kg) through the tail vein (suspended in 400 μl PBS). On day 28, the tibiae were harvested for micro-CT and histomorphometric analysis.

### Micro-CT and bone mineral density analysis

Rat tibiae were imaged using a computerized tomography (micro-CT) (VivaCT40; Scanco Medical, Switzerland) with a resolution of 10.2 μm (Additional file 1). To analyze the trabecular bone of tibial metaphysis, a region below the growth plate from 2.0 to 4.0 mm was used for quantification (cortical bone was not included). The morphology of the trabecular bone in the tibial metaphysis was measured using Analyze 12.0 (registered, Center of Biomedical Analysis, Third Military Medical University). Bone mineral density (BMD), bone volume to total tissue volume ratio (BV/TV), connective density (Conn.D), trabecular number (Tb.N), and trabecular thickness (Tb.Th) were assessed.

### Isolation and culture of rat BM-MSCs

Sprague-Dawley rats (80 g, female) were sacrificed by cervical dislocation, and femurs and tibias were separated from hind limbs. The ends of the femur or tibia were removed, and the bone marrow was flushed out with 1 ml DMEM/F12 medium. The bone marrow was repeatedly washed to generate a single-cell suspension that was centrifuged at 1000 rpm for 5 min. The supernatant was removed, and cells were washed with DMEM/F12 and centrifuged for an additional 5 min. Finally, the supernatant was removed, and cells were resuspended in DMEM/F12 medium containing 10% fetal bovine serum (FBS) and 1% penicillin-streptomycin. Cells isolated from one hind limb were plated in a 25-cm^2^ dish and incubated at 37 °C with 5% CO2, which was defined as passage 0 (P0). After 24 h, cells were washed with PBS twice to remove non-adherent cells. When cell confluency was greater than 90%, the cells were secondarily cultured, and the passage number was increased by one. Cells from passages 2 and 3 were used to collect exosomes.

### Isolation and characterization of exosomes

Isolation and purification of exosomes involved several centrifugation and ultracentrifugation (Himac cp80wx/P70A-980, Hitachi Limited, Tokyo, Japan) steps as described previously [33–35]. Briefly, exosomes from bovine were removed by ultracentrifugation at 100,000 × *g* at 4 °C for 16 h to generate exosome-free serum. P2 or P3 BM-MSCs were cultured in exosome-free medium. Then, the supernatant was collected after 48 h. To isolate and purify exosomes, the medium was centrifuged at 300 × *g* for 15 min and 2000 × *g* for 15 min to remove cells and cell debris. Then, the supernatant was transferred to a 35-ml ultracentrifuge tube and ultracentrifuged at 4 °C for 70 min at a speed of 100,000 × *g*. After the first ultracentrifugation, each tube was washed with 5 ml PBS and then filtered through a 0.22-μm membrane filter. Then, the exosomes were collected by another 100,000 × *g* ultracentrifugation for 70 min at 4 °C. The final pellet (obtained from approximately 500 ml medium) was resuspended in 200 μl PBS and stored at − 80 °C. The protein concentration of the collected exosomes was measured using a BCA protein assay kit (Beyotime, China). The collected exosomes were dehydrated in absolute ethanol for 10 min and collected on a carbon-stable Format grid. The gate was compared with 1% phosphotungstic acid for 1 min, then the air-dried exosome-containing grids were observed by transmission electron microscopy (JEM-1400PLUS, Japan) at 100 KV.

### Labeling and tracking of exosomes in BM-MSCs

Labeling and tracking of exosomes in BM-MSCs was performed as described previously [33]. According to the manufacturer’s protocol, BM-MSCs and exosomes were labeled with CM-Dio(3,3′-dioctadecyloxacarbocyanine perchlorate) and CM-DiI(1,1′-dioctadecyl-3,3,3′,3′-tetramethylindocarbocyanine perchlorate) (Beyotime Biotechnology, Haimen, China), respectively, and then cultured in the dark at 37 °C for 30 min. To remove unbound dye, exosomes and BM-MSCs were washed with PBS and then centrifuged at 100,000 × *g* at 4 °C for 70 min and 800 × *g* at room temperature for 5 min, respectively. Finally, exosomes and BM-MSCs were mixed together and incubated at 37 °C for 24 h. The uptake of exosomes was observed by fluorescence microscopy (Leica, Weltzlar, Germany), and images were analyzed using Leica Application Suite Advanced Fluorescence (LAS AF) software.

### Irradiation of cells

When passage 3 BM-MSCs reached 80–90% confluency, they were divided into three groups: control group, BM-MSCs not exposed to irradiation; 6 Gy group, BM-MSCs receive 6 Gy irradiation; 6 Gy+Ex group, BM-MSCs receive 6 Gy irradiation and cocultured with exosomes immediately (100 μg/ml). BM-MSCs were incubated at 37 °C with 5% CO2 for a specified time, and cells were collected or used for other experiments. The irradiation was performed using a Co-60 irradiator at a rate of 0.56 Gy/min (Radiation Center, Army Medical University).

### Colony formation assay

BM-MSCs were seeded in six-well plates (1 × 10^4^ cells per well). After 14 days, cells were washed with PBS twice and fixed with 4% formaldehyde at room temperature for 20 min. Cells were stained with 2% crystal violet for 10 min, and then, the unconjugated dyes were removed. A stained colony composed of 50 or more cells were counted as a colony-forming unit (CFU).

### SA-β-gal staining

SA-β-gal staining was performed using an SA-β-gal staining kit (Beyotime Biotechnology, Haimen, China) according to the manufacturer’s protocol. Briefly, cells were incubated at 37 °C with 5% CO^2^ for 24 h after irradiation, then cells were seeded in 12-well plates (5 × 10^3^ cells per well) for an additional 24 h. Cells were washed with PBS twice and fixed with 4% formaldehyde at room temperature for 20 min. Then, cells were stained with X-gal solution for 24 h at 37 °C (no CO^2^). SA-β-gal-positive cells were observed using a light microscope (Leica, Weltzlar, Germany), and the percentage of positive cells in ten random fields was calculated. The results are expressed as the mean of triplicates with SD.

### Immunofluorescence

After treatment, cells were fixed with 4% formaldehyde for 20 min at room temperature. Cells were permeabilized by Triton X-100 and subjected to goat serum blocking (Beyotime Biotechnology, Haimen, China). Cells were then incubated with primary antibodies against γ-H2AX (1:100 dilution) (Santa Cruz, CA, USA) and β-catenin (1:200 dilution) (Beyotime Biotechnology, Haimen, China) overnight at 4 °C. Cells were rinsed with PBS twice and then incubated in the dark with respective secondary antibodies for 60 min and DAPI for 5 min (Beyotime Biotechnology, Haimen, China). Using a fluorescence microscope (Leica, Weltzlar, Germany), the number of γ-H2AX foci per cell was quantified. Then, β-catenin fluorescence was measured by laser confocal microscopy (Olympus, Tokyo, Japan).

### Detection of reactive oxygen species (ROS)

Production cellular ROS was measured utilizing a Reactive Oxygen Species Assay kit (Beyotime Biotechnology, Haimen, China) according to the manufacturer’s instructions. Briefly, cells were seeded in a six-well plate (1 × 10^5^/well) and exposed to different treatments. The cells were washed with PBS twice and incubated with 2′,7′-dichlorodihydrofluorescein diacetate (DCF-DA) (10 μM) for 30 min at 37 °C. Then, the medium was removed, and cells were washed with PBS. Cells were collected, and the fluorescence intensity of each sample was examined by flow cytometry (Additional file 2). Similarly, intracellular ROS fluorescence was observed using a fluorescence microscope (Leica, Weltzlar, Germany).

### Quantitative real-time PCR

Total RNA was extracted using the Trizol reagent (Invitrogen, Carlsbad, CA, USA) according to the manufacturer’s instructions. First-strand cDNA was synthesized from 2 μg of RNA using the PrimeScript RT Master Mix Kit (Takara Bio, Shiga, Japan). qPCR was performed in triplicate in 10-μl reactions containing SYBR Premix Ex Taq II (Takara Bio, Shiga, Japan). The reaction protocol was as follows: heating at 95 °C for 5 min followed by 40 rounds of amplification (30 s at 95 °C, 30 s at 59 °C, and 30 s at 72 °C). The expression of each gene was normalized to β-actin expression.

RT-PCR primers were as follows: β-actin: 5′-GCAGATGTGGATCAGCAAGC-3′, 3′-AGAAAGGGTGTAAAACGCAGC-5′; Ctnnb1 (β-catenin): 5′-ACTCCAGGAATGAAGGCGTG-3′, 3′-GAACTGGTCAGCTCAACCGA-5′; Ebf1: 5′-AGGGCTAGGAGGCTTGACC-3′, 3′-CCGTCGTCCATCCTTCACTC-5′; OPG: 5′-TGTCCCTTGCCCTGACTACT-3′, 3′-CACATTCGCACACTCGGTTG-5′; PPARγ: 5′-TGTTATGGGTGAAACTCTGGGA-3′, 3′-TAGGCAGTGCATCAGCGAAG-5′; RUNX2: 5′-CCTTCCCTCCGAGACCCTAA-3′, 3′-ATGGCTGCTCCCTTCTGAAC-5′.

### Western blotting analysis

Cells were harvested and lysed in Western and IP buffer (Beyotime Biotechnology, Haimen, China), and total protein concentrations were determined using the BCA Protein Assay Kit (Beyotime Biotechnology, Haimen, China). Equal amounts of cell lysates were loaded and separated on a 12% SDS-PAGE gel and transferred to 0.22-μm PVDF membranes (Millipore Billerica, MA, USA). The membranes were blocked with QuickBlock™ Blocking Buffer (Beyotime Biotechnology, Haimen, China) for 15 min at room temperature and then incubated with primary antibodies overnight at 4 °C. Then, membranes were incubated with HRP-conjugated secondary antibodies for 1 h at room temperature.

The following primary antibodies were used for blotting. Mouse antibodies against p21, p16, p53, Rb, Runx2, PPARγ, γ-H2AX, CD63, Tsg101, and CD81 were purchased from Santa Cruz Biotechnology (Santa Cruz, CA, USA). Mouse antibodies against β-actin and rabbit antibodies against β-catenin were purchased from Beyotime Biotechnology (Beyotime Biotechnology, Haimen, China). Rabbit antibodies against calnexin, SOD1, SOD2, and catalase were purchased from Abcam (Abcam, Cambridge, UK). The specific information of antibodies is listed in Table S1.

### Osteogenic and adipogenic differentiation

To induce differentiation, cells were seeded in 12-well plates. When cell confluency reached 60–70%, the cells were incubated with rat mesenchymal stem cell osteogenic differentiation medium (Cyagen Biosciences, Guangzhou, China) for 14 days or adipogenic differentiation medium for 15 days. After the induction of differentiation, cells were fixed with 4% formaldehyde for 20 min at room temperature. Cells were washed with PBS twice and stained with alizarin red S or Oil red O for 30 min. The stained cultures were visualized under a light microscope (Leica, Weltzlar, Germany).

### Histology and immunohistochemistry

Left rat tibiae were fixed in 4% formaldehyde for 24 h and decalcified for 21 days with 10% EDTA. Then, the tibiae were embedded in paraffin and sectioned coronally at a thickness of 4 μm. The sections were stained with hematoxylin and eosin (H&E). For immunohistochemistry staining, the endogenous peroxidase activity of the sections was quenched using 2.5% (*v*/*v*) hydrogen peroxide in distilled water and then performing pepsin-mediated antigen retrieval (ZSGB-BIO, Beijing, China). After blocking with goat serum, the sections were incubated with primary antibodies against β-catenin (1:200 dilution) overnight at 4 °C. The sections were then incubated with HRP-conjugated secondary antibodies at room temperature. The immunoreactive signals were detected with a DAB Kit (ZSGB-BIO, Beijing, China). Images were obtained with a light microscope (Leica, Weltzlar, Germany).

## Results

### Identification of exosomes and uptake by BM-MSCs

**Fig. 1:**
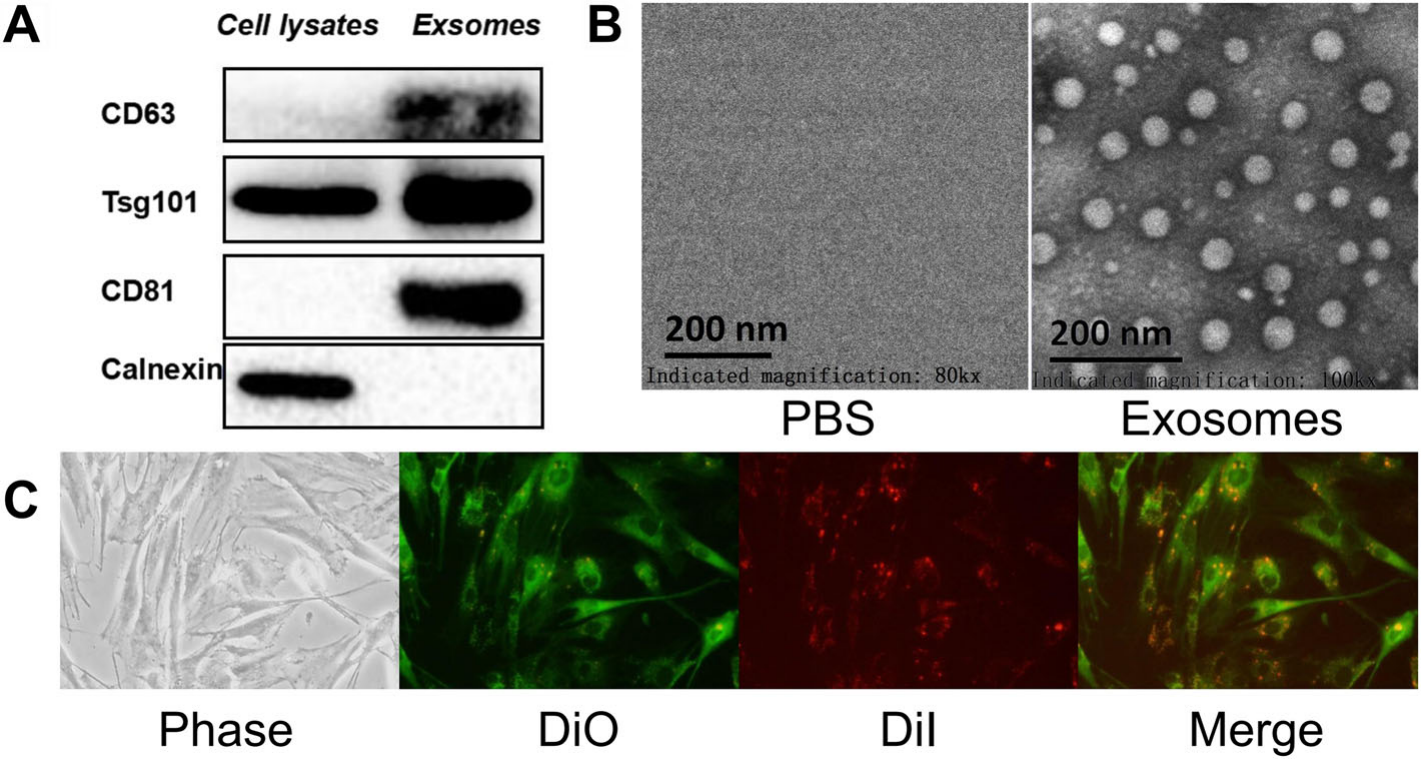
Identification of exosomes and uptake of exosomes by BM-MSCs. **a** Western blot analyses of exosomal markers CD63, CD81, Tsg101, and negative protein calnexin in BM-MSC lysates and BM-MSC-derived exosomes. **b** Characterization of BM-MSC-derived exosomes by transmission electron microscopy. PBS was used as control. Exosomes were round or oval with a diameter of approximately 40–100 nm. Scale bar = 200 nm. **c** Uptake of CM-DiI (red)-labeled exosomes in CM-DiO (green)-labeled BM-MSCs were observed using a fluorescence microscope after 24 h. Abbreviations: PBS, phosphate-buffered saline; BM-MSCs, bone marrow mesenchymal stem cells

Stem cell surface markers, morphology and multipotency of BM-MSCs were measured (Additional file 2: Figure S2). Exosomes isolated from BM-MSCs were identified by Western blot and transmission electron microscopy. Western blot showed that exosomes highly expressed exosome-positive markers CD63, CD81, and Tsg101, but did not express calnexin, a negative marker of exosomes (Fig. 1a). Under transmission electron microscopy, the exosomes were round or oval with a diameter of approximately 40–100 nm and a bilayer membrane structure (Fig. 1b). To observe the uptake of the exosomes by BM-MSCs, BM-MSCs and exosomes were labeled with fluorescent carbocyanine dyes CM-Dio (green) and CM-Dil (red), respectively. After 24 h of coincubation, we observed the distribution of exosomes in BM-MSCs using a fluorescence microscope, suggesting that the exosomes were successfully absorbed by BM-MSCs (Fig. 1c).

### BM-MSC and exosome transplantation alleviate bone loss induced by irradiation

**Fig. 2:**
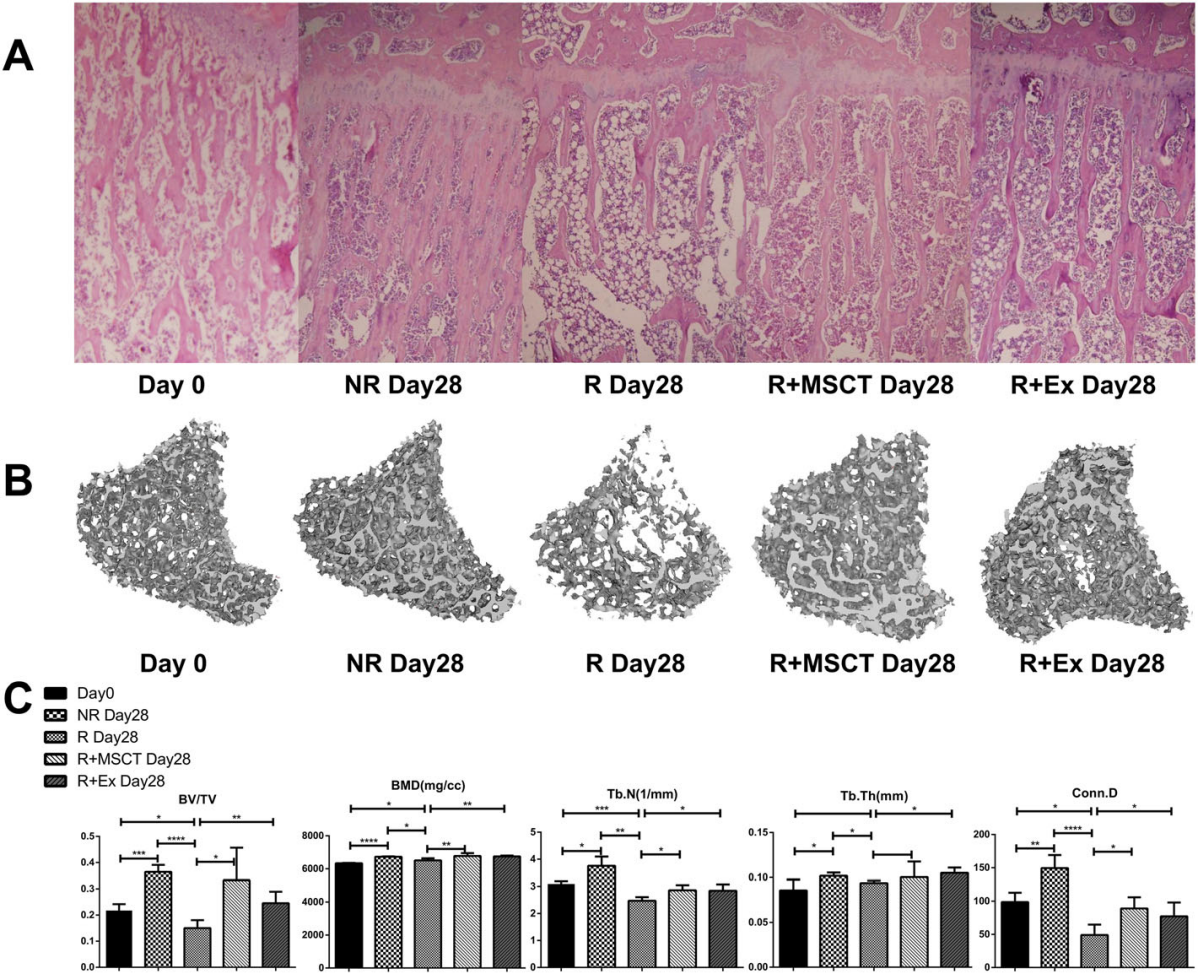
BM-MSC and exosome transplantation alleviates bone loss induced by irradiation. **a** HE staining of rat left tibia in each group. **b** 3D micro-CT image of the target region (a region below the growth plate from 2.0 to 4.0 mm). **c** Bone microarchitecture analysis of target region in the proximal of left tibia. Abbreviations: BV/TV, bone volume to total tissue volume ratio; BMD, bone mineral density; Conn. D, connective density; Tb. N, trabecular number; Tb. Th, trabecular thickness. Data represent the mean ± SD (*n* = 5 independent experiments, *t* test).*p < 0.05, **p < 0.01, ***p < 0.001, ****p < 0.0001. Abbreviations: day 0, rats with no treatment and sacrificed on day 0; day 28, rats with no treatment and sacrificed on day 28; R day 28, rats received 16 Gy irradiation on day 0 and sacrificed on day 28; R+MSCT day28, rats received 16 Gy irradiation and MSC transplantation and sacrificed on day 28; R+Ex day 28, rats received 16 Gy irradiation and exosome transplantation and sacrificed on day 28

To simulate clinical radiotherapy, 3-month-old female rats were irradiated with a 16-Gy dose on the left knee. To prevent irradiating other parts, we used lead bricks to block the other areas of rats. After irradiation, rats were divided into five groups (*n* = 6). MSCs and exosomes were transplanted immediately in the R+MSCT and R+Ex groups, respectively. Unlike human beings, the growth plate of the rat does not close in adulthood, so we used it as a reference object for measuring bone histomorphometry parameters. On day 28 after irradiation, the tibiae were harvested for micro-CT and histomorphometric analysis. HE staining revealed a significant decrease in bone volume and trabecular number in the R group but an increase in marrow adiposity (Fig. 2a). However, the R+MSCT and R+Ex group exhibited remarkable improvement, and similar results were observed by 3D reconstruction of CT scan (Fig. 2b). These findings indicate that radiation can shift the bone remodeling balance to activate resorption, and MSC or exosome transplantation can alleviate radiation-induced bone loss. To quantify these changes, we used Analyze 12.0 software for bone microarchitecture analysis. As shown in Fig. 2c, bone volume fraction (BV/TV) in the day 28 group increased by approximately 67.6% compared with that in the day 0 group. In contrast, values in the R group decreased by approximately 30.9%. However, in the R+MSCT and R+Ex groups, BV/TV increased by 53% and 13%, respectively, and the difference was statistically significant compared with the R group. In the comparison of BMD, the data of each group were also significantly different. In the comparison of trabecular number (Tb.N), trabecular thickness (Tb.Th), and connective density (Conn.D), values in the R group decreased significantly compared with those in the day 28 group. However, values in the R+MSCT and R+Ex groups were not significantly increased or even decreased compared with those in the day 0 group. However, these values were significantly improved compared with those of the R group, and many data exhibited significant differences (Fig. 2c).

### Exosomes alleviate radiation-induced oxidative stress in BM-MSCs

**Fig. 3:**
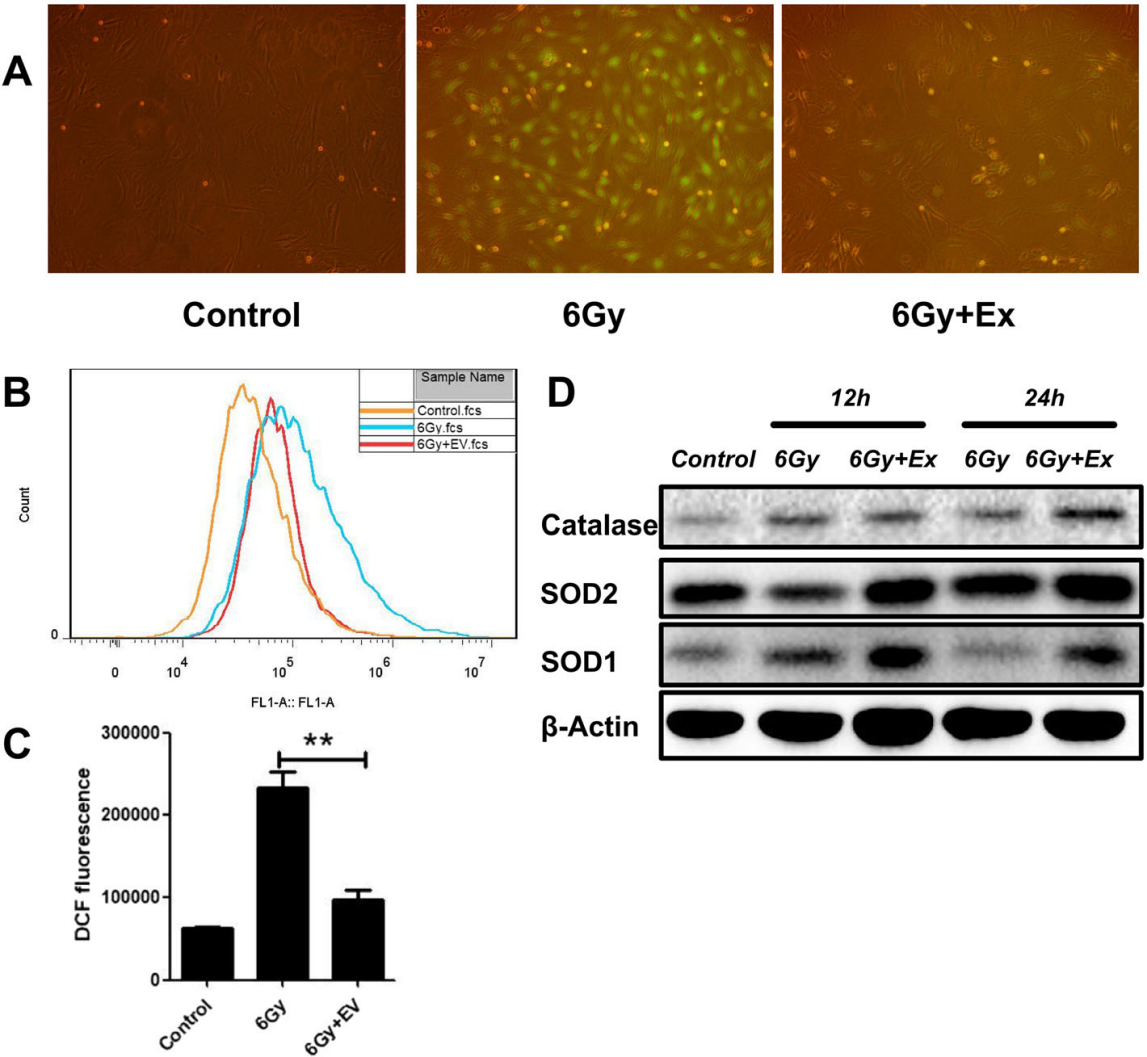
Exosomes alleviate radiation-induced oxidative stress in BM-MSCs. **a** ROS production in irradiated and exosomes cocultured with BM-MSCs were detected by DCF fluorescence after 24 h. **b** ROS levels were assessed by DCF fluorescence intensity via flow cytometry. Relative DCF fluorescent values revealed reduced ROS production in exosomes cocultured with BM-MSCs. **c** Quantitative analysis of fluorescence intensity. Data are presented as the mean ± SD. **p < 0.01. **d** Western blot analysis of antioxidant protein catalase, SOD2, and SOD1. Abbreviations: 6 Gy, BM-MSCs received 6 Gy gamma irradiation; 6 Gy+Ex, BM-MSCs received 6 Gy gamma irradiation then cocultured with 100 μg/ml exosomes

Following irradiation, increased reactive oxygen species in the mesenchymal stem cells can cause cell damage. MSC-derived exosomes reduce intracellular reactive oxygen species (ROS) to protect cells from damage both in vivo and in vitro. Therefore, we hypothesized that exosomes could reduce cellular damage by reducing reactive oxygen species after irradiation. To test this hypothesis, we assessed reactive oxygen species using DCF-DA 24 h after exosome and BM-MSC coincubation. Under fluorescence microscopy, reactive oxygen species in irradiated BM-MSCs decreased significantly after coincubation with exosomes (Fig. 3a). Further measurements of reactive oxygen species by flow cytometry revealed that DCF fluorescence in BM-MSCs decreased significantly after exosome treatment (Fig. 3b, c). Then, we used Western blot to detect the expression of antioxidant-related proteins. We found that the expression of antioxidant proteins, such as catalase, SOD1, and SOD2, increased after coincubation with exosomes at 12 h and 24 h. These results suggest that coincubation with exosomes can enhance the antioxidant capacity of irradiated BM-MSCs (Fig. 3d).

### Exosomes accelerate DNA repair in BM-MSCs after irradiation

**Fig. 4:**
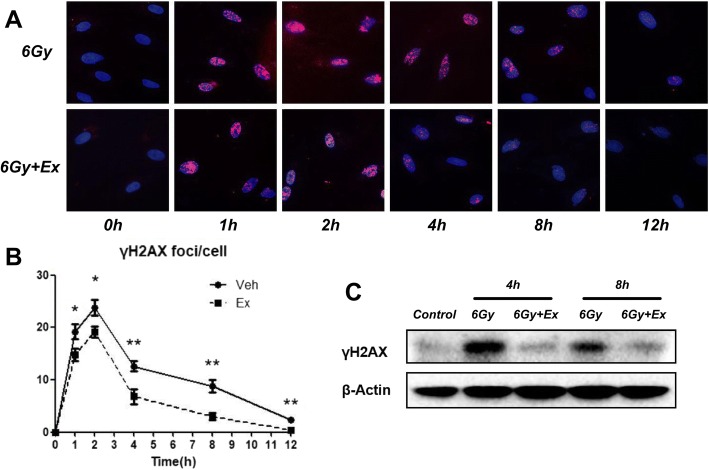
Exosomes accelerate DNA repair in BM-MSCs after irradiation. **a** Immunofluorescence staining of γ-H2AX in BM-MSCs after irradiation cocultured with PBS or exosomes at different time points. **b** Quantitative analysis of γ-H2AX foci per cell. The data are presented as the mean ± SD. *p < 0.05, **p < 0.01. **c** Western blot analysis of γ-H2AX

Another major form of damage to cells caused by irradiation is the DNA double-strand break (DSB), which will lead to cell senescence and mitotic catastrophe if the damage is misrepaired. γ-H2AX, which is also known as the phosphorylated histone H2A variant, is an important protein marker for detecting DSB. To verify whether exosomes can accelerate DNA damage repair following irradiation, we used immunofluorescence and Western blot to detect γ-H2AX. We found that the expression of γ-H2AX in BM-MSCs increased rapidly after irradiation, peaked at approximately 2 h, gradually decreased after 4 h, and was almost not detected after 12 h (Fig. 4a, b). Compared with irradiation alone, γ-H2AX expression in BM-MSCs co-incubated with exosomes was reduced at all time points, and the difference in γ-H2AX foci per cell was statistically significant (Fig. 4a, b). In addition, the expression levels of γ-H2AX in BM-MSCs decreased significantly at 4 and 8 h after coculture with exosomes (Fig. 4c). These results provide evidence that exosomes can effectively promote DNA damage repair in BM-MSCs after irradiation.

### Exosomes rescued the inhibition of proliferation and decreased the senescence-associated protein expression in BM-MSCs after irradiation

**Fig. 5:**
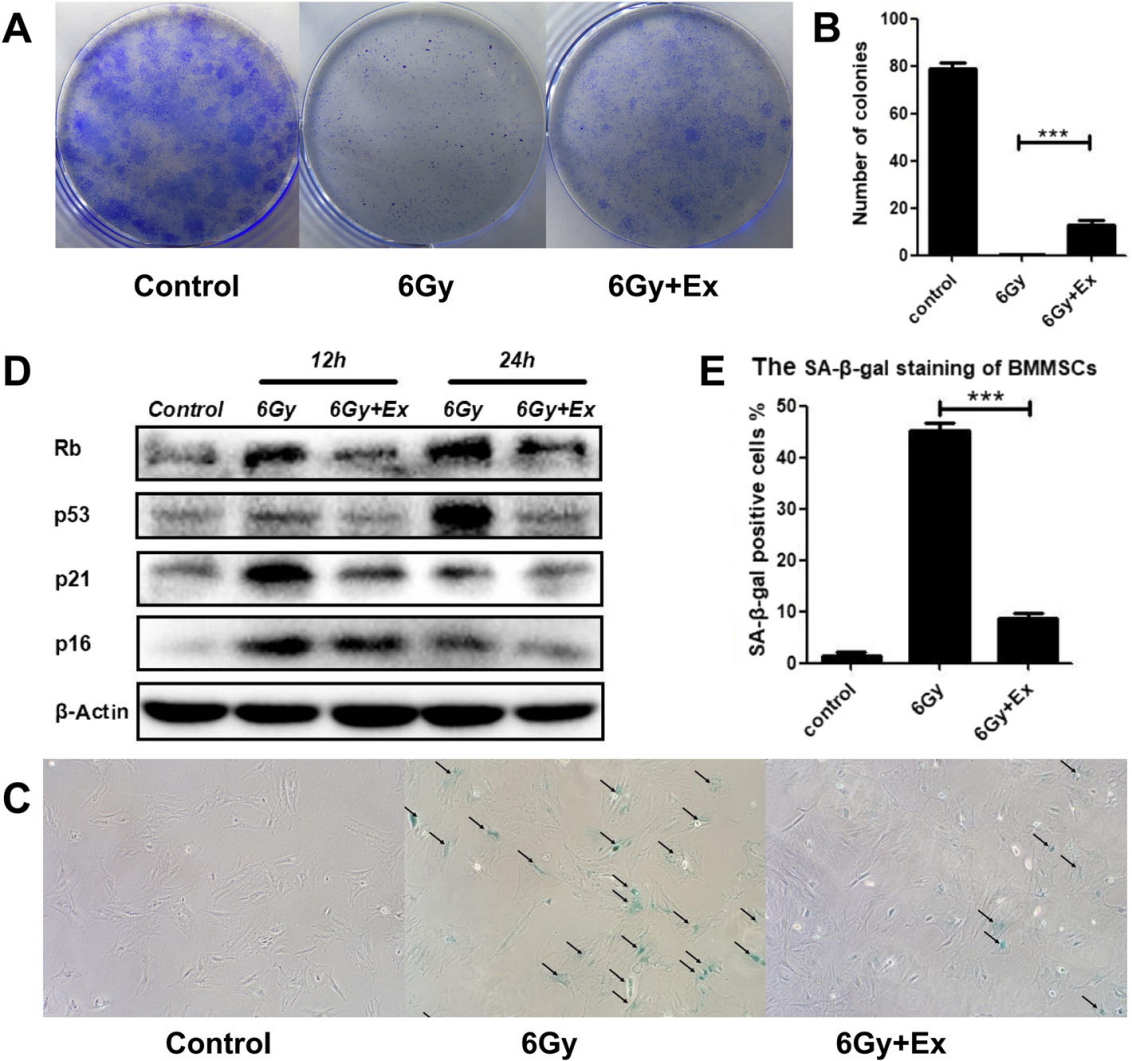
Exosomes rescued the inhibition of proliferation and decrease the senescence-associated protein expression of BM-MSCs after irradiation. **a** Colony-forming ability of BM-MSCs after irradiation. Colony formation was assessed by staining with crystal violet. **b** Quantitative analysis of colony-forming units. Data are presented as the mean ± SD (*n* = 3 independent experiments, *t* test). ***p < 0.001. **c** Western blot analysis of senescence-associated proteins, including Rb, P53, P21, and P16. **d** Percentage of SA-β-gal-positive cells under different treatments. Data are presented as the mean ± SD (*n* = 10 independent experiments, t test). ***p < 0.001. **e** Senescence-associated β-galactosidase (SA-β-gal) staining

Given that exosomes can promote the DNA damage repair of BM-MSCs after irradiation, exosomes may also reverse cell proliferation inhibition and alleviate cell senescence. To verify this hypothesis, we cocultured irradiated BM-MSCs with exosomes for 24 h, seeded cells into six-well plates (1 × 10^4^ cells per well), and cultured cells for 14 days. Stained colonies of 50 or more cells were counted as a colony-forming unit (CFU). We found that after 6 Gy irradiation, no colonies formed in the 6 Gy group. Although the CFU number was still reduced compared with the control group, the CFU number in the 6 Gy+Ex group was significantly increased compared with that in the 6 Gy group, and the difference was statistically significant (Fig. 5a, b). In addition, Western blot also demonstrated that after being cocultured with exosomes, the expression of aging-related proteins, including Rb, p53, p21, and p16, was decreased at 12 and 24 h compared with BM-MSCs that were only irradiated (Fig. 5c). SA-β-gal staining was highly consistent with these results (Fig. 5d, e). Thus, coincubation with exosomes can decrease the senescence-associated protein expression and partially restore cell proliferation after irradiation.

### Exosomes restored the differentiation potential of irradiated BM-MSCs

**Fig. 6:**
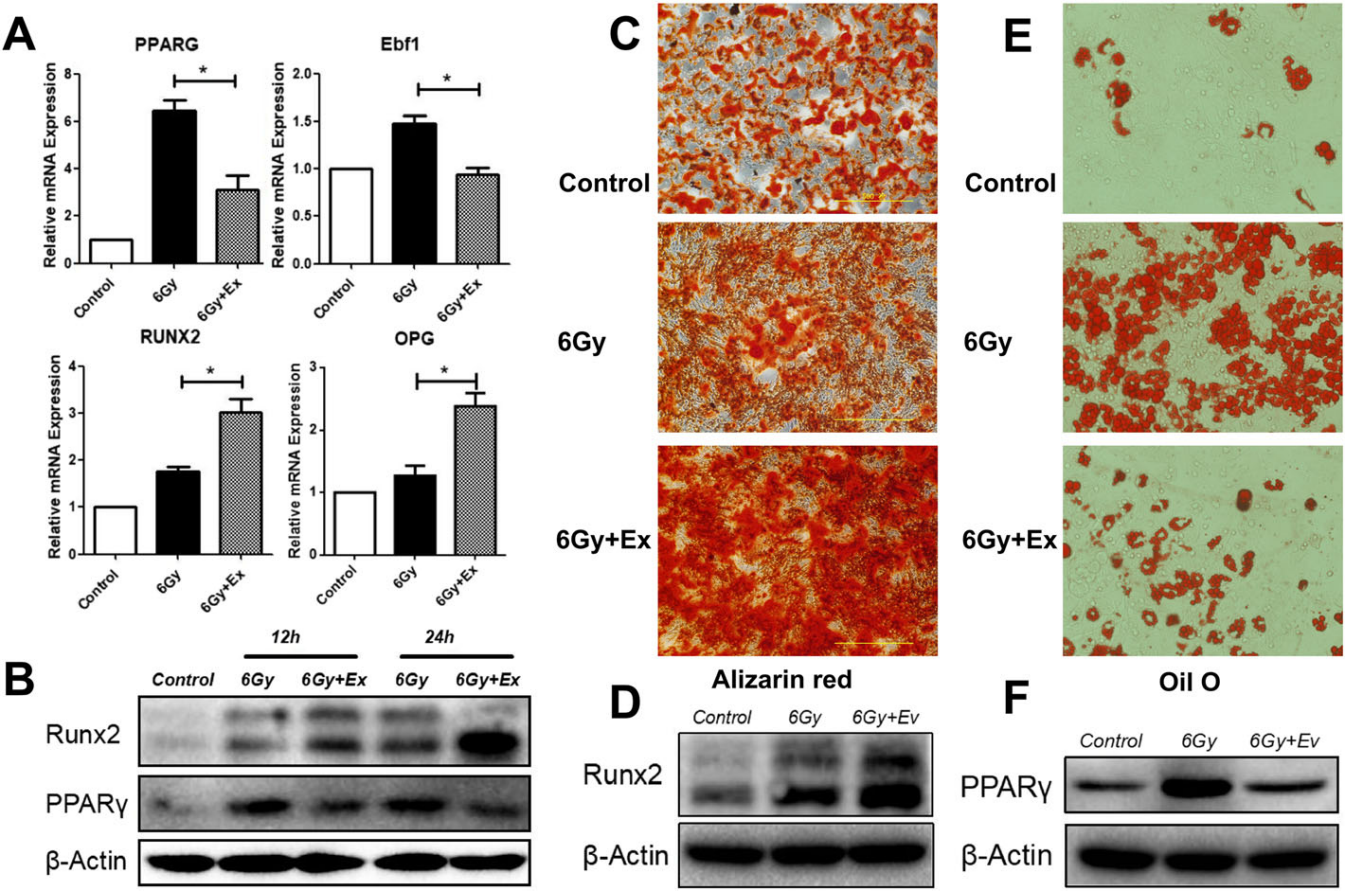
Exosomes restored the balance between adipogenic and osteogenic differentiation of irradiated BM-MSCs. **a** RT-PCR analyses of adipogenic and osteogenic genes, including PPARγ, Ebf1, Runx2, and OPG. Gene expression was normalized to β-actin and control. Data are presented as the mean ± SD (*n* = 3 independent experiments, *t* test). *p < 0.05. **b** Western blot analysis of PPARγ and RUNX2. **c** BM-MSCs were stained with alizarin red after 14 days of osteogenic induction. **d** Western blot analysis of RUNX2 after 14 days of osteogenic induction. **e** BM-MSCs were stained with oil O after 15 days of adipogenic induction. **f** Western blot analysis of PPARγ after 15 days of adipogenic induction

Irradiation can lead to apoptosis of osteoblasts and osteocytes, disrupt the differentiation potential of BM-MSCs, and eventually reduce bone mass. After irradiation, exosome transplantation reduced bone loss in the tibiae of rats, indicating that it can restore the balance between adipogenic and osteogenic differentiation of BM-MSCs. To test this hypothesis, we measured adipogenic- and osteogenic-related mRNA and protein expression in BM-MSCs 24 h after irradiation. The expression of adipogenesis-related mRNA PPARγ [36] and Ebf1 [37] and osteogenesis-related mRNA RUNX2 [38] and OPG [39] in the 6 Gy group increased after irradiation, but the proportion of adipogenesis-related mRNA increased to levels greater than that of osteogenesis-related mRNA (Fig. 6a). Compared with the 6 Gy group, adipogenesis-related mRNA in the 6 Gy+Ex group was significantly reduced, while osteogenesis-related mRNA increased. The differences were statistically significant (Fig. 6a). Similar results were noted for protein expression (Fig. 6b). Expression of mRNA and protein suggested that BM-MSCs start differentiation after irradiation, but the differentiation exhibits a tendency such that differentiation towards adipocytes exceeds that to osteoblasts.

To observe the effects of irradiation and exosomes on BM-MSC differentiation, cells were cultured in osteogenic or adipogenic differentiation medium. After 14 days of osteogenic induction, the 6 Gy+Ex group exhibited the highest proportion of calcium deposition, and the 6 Gy group also exhibited higher calcium deposition compared with the control group (Fig. 6c). In addition, Western blot analysis also showed the same trend in detecting the expression of Runx2 (Fig. 6d). However, after 15 days of adipogenic induction, the 6 Gy group exhibited the highest oil O staining (Fig. 6e). Although the 6 Gy+Ex group exhibited increased rates of oil staining compared with the control group, the levels are still considerably reduced compared with that of the 6 Gy group (Fig. 6e). Similar results were also been found in Western blot analysis of PPARγ (Fig. 6f). These results suggested that BM-MSCs start differentiation after radiation, but the differentiation tendency towards adipocytes exceeds that to osteoblasts, and coculture with exosomes could restore the differentiation potential of irradiated BM-MSCs.

### Exosomes activate wnt/β-catenin pathway of BM-MSCs after irradiation

**Fig. 7:**
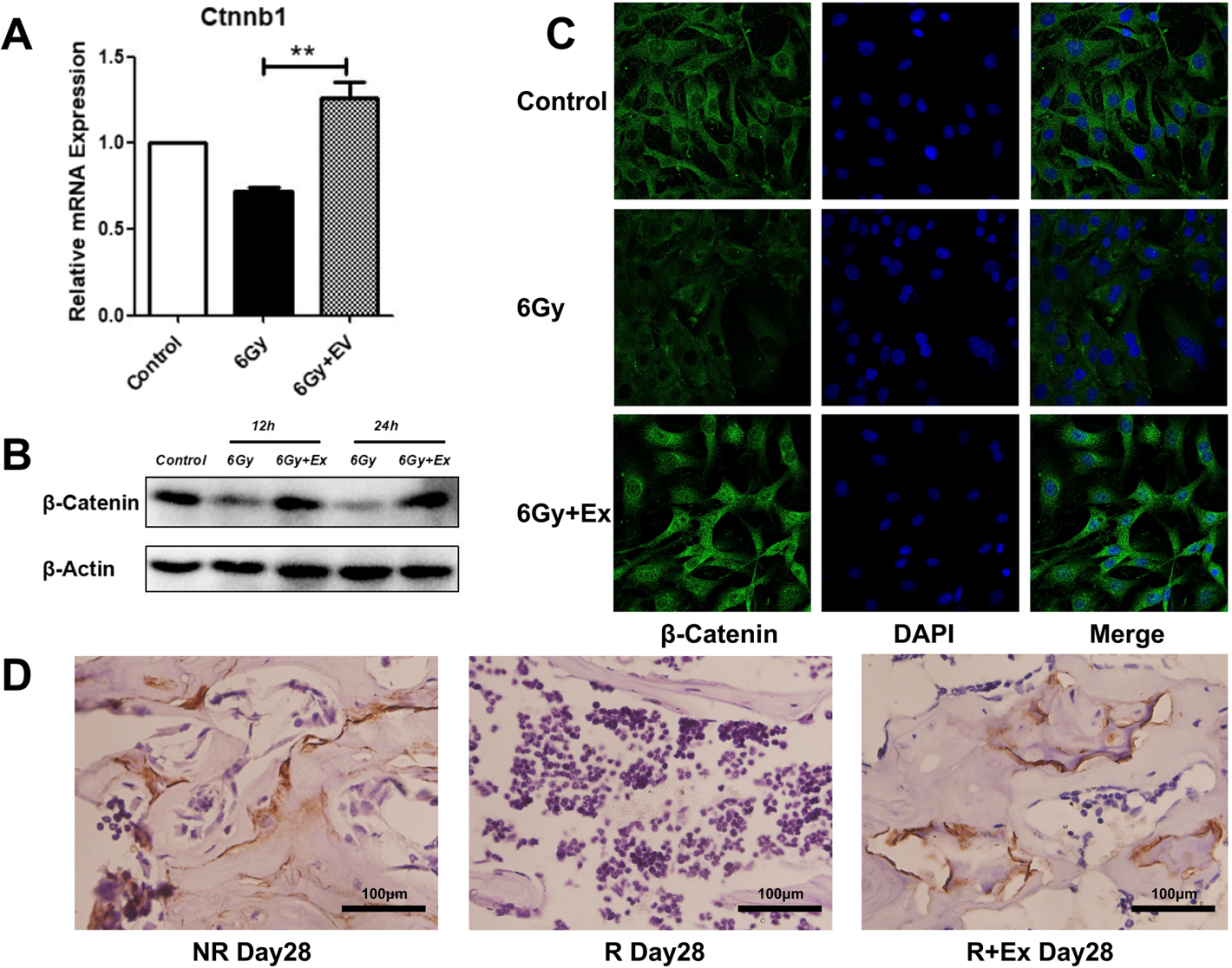
Exosomes activate the wnt/β-catenin pathway of BM-MSCs after irradiation. **a** RT-PCR analyses of Ctnnb1. Data are presented as the mean ± SD (*n* = 3 independent experiments, *t* test). *p < 0.05. **b** Western blot analysis of β-catenin. **c** Immunofluorescence staining of β-catenin in BM-MSCs. **d** Immunohistochemical analysis of β-catenin in rat left tibia. Scale bars, 100 μm

The Wnt/β-catenin pathway is a classical pathway involved in bone metabolism regulation. When activated, it can promote osteoblast precursor cells to transform into osteoblasts. We hypothesized that coculture with exosomes can activate the Wnt/β-catenin pathway of BM-MSCs after irradiation. We found that β-catenin mRNA and protein expression in the 6 Gy group was reduced compared with that in the control group, and the expression of β-catenin increased obviously in the 6 Gy+Ex group (Fig. 7a). Using immunofluorescence analysis, we also found that the fluorescence intensity of β-catenin in the 6 Gy+Ex group is obviously increased compared with that in the 6 Gy group (Fig. 7b). A similar result was also obtained in the immunohistochemistry analyses (Fig. 7c). These results indicate that after coculture with exosomes of irradiated BM-MSCs, the Wnt/β-catenin pathway was activated and promoted osteogenesis, thus reducing the decrease in bone mass induced by irradiation.

## Discussion

Mesenchymal stem cell transplantation is effective in the treatment of various diseases, including cardiovascular diseases [40, 41], liver diseases [42], and brain injury [43], both in experimental and clinical research. The therapeutic effects of transplanted MSCs can be achieved by homing directly to injured tissues or secreting paracrine factors. Recently, MSC-derived extracellular vesicles (EVs), including exosomes and microvesicles (MVs), have been studied given their role in MSC-based cell therapy. Exosome/microvesicles contain mRNA, microRNA, and protein, which are involved in intercellular communication, cell signal transduction, and alterations in the metabolism of cells or tissues in the body over short or long distances [24].

Recent studies have demonstrated that BM-MSC-derived exosomes play an important role in the treatment of bone loss-related diseases. Liu et al. [22] reported that exosomes secreted from transplanted MSCs rescue the function of the osteoporotic phenotype of recipient BM-MSCs and improve osteopenia via epigenetic regulation. Furthermore, Liu et al. [44] found that transplantation of MSC-derived exosomes exerts a preventive effect on osteonecrosis of the femoral head by promoting local angiogenesis and preventing bone loss. In this study, we established a radiation-induced bone injury model in the left tibiae of rats. After irradiation, we treated the rats differently, and we found that transplantation of MSC or exosomes could alleviate radiation-induced bone loss. Based on our results, we hypothesize that exosomes may have similar protective and repairing properties as MSC transplantation in radiation-induced bone loss. Compared with MSC transplantation, exosome therapy is preferable given the reduced immune responses, increased safety, and ease of storage, transportation, and management.

Mesenchymal stem cell damage is an important pathological mechanism of radiation-induced bone loss [9, 12, 45]. Exposure to irradiation causes BM-MSCs to generate reactive oxygen species, and excessive ROS leads to DNA damage, such as DNA double-strand breaks (DSBs) [9, 12, 45]. Therefore, clearing up reactive oxygen species and reducing DNA damage are potential treatments for irradiation-induced bone loss. Recent studies have demonstrated that MSCs-derived exosomes reduce oxidative stress and alleviate DNA damage. Yan et al. [33] reported that MSC-derived exosomes promote hepatic oxidant injury through the delivery of GPX1. Lin et al. [29] reported that adipose-derived MSC-derived exosomes could accelerate DNA repair and protect kidneys from ischemia-reperfusion injury. Our results demonstrated that BM-MSC-derived exosomes can reduce irradiation-induced oxidative stress and promote the expression of antioxidant protein. Furthermore, we also observed that BM-MSC-derived exosomes can alleviate irradiation-induced DNA damage as determined by γ-H2AX staining and Western blot.

Whether irradiation affects the viability of MSCs remains controversial, but many studies indicate that radiation can hinder the capacity of MSCs to proliferate and strongly increase cellular senescence. Danielle E Green et al. reported that irradiation exposure destroys bone marrow stem cell pools and hypothesized that stem cell pool recovery enables the prompt repair of the skeletal system and ultimately reduces the susceptibility to fractures. Moreover, radioresistant MSCs exhibited strong beta-galactosidase activity and increased the expression of cell cycle-dependent kinase inhibitor 2A (P16-IK4A) at late time points after 60-Gy doses of irradiation [46, 47]. The senescence induction of MSCs is mediated by retinoblastoma protein, RB, cyclin-dependent kinase inhibitor 1A(p21), and tumor suppressor p53 [48, 49]. Inhibition of proliferation and premature senescence of BM-MSCs induced by irradiation reduced functional and viable MSCs in bone marrow [9]. As exosomes can reduce oxidative stress and alleviate DNA damage, we hypothesize that exosomes can also rescue the inhibition of proliferation and alleviate cellular senescence in BM-MSCs after irradiation. In this study, we found that BM-MSC-derived exosomes could partially rescue the inhibition of proliferation, as determined by CFU assays. We also found that BM-MSC-derived exosomes could alleviate cell senescence of BM-MSCs following irradiation, as detected by SA-β-gal staining and Western blotting.

Unlike BM-MSCs, osteocytes and osteoblasts are highly sensitive to radiation, and low doses of radiation can induce apoptosis [50, 51]. Unfortunately, following irradiation, BM-MSCs seem to preferentially differentiate into adipocytes rather than osteoblasts [6, 11]. As a result, the apoptotic osteocytes and osteoblasts cannot be replenished in a timely manner, which ultimately hinders proper bone formation and leads to bone loss-related diseases. Therefore, restoring the balance between adipogenic and osteogenic differentiation of irradiated BM-MSCs is the key to treat radiation-induced bone loss. To determine whether BM-MSC-derived exosomes can reconstruct the differentiation potential of radiated BM-MSCs, we examined the expression of mRNA and protein related to osteogenesis and adipogenesis in irradiated BM-MSCs. We found that irradiated BM-MSCs treated with exosomes exhibited reduced adipogenic gene expression and increased osteogenic gene expression compared with those treated with radiation only. In addition, we also observed that after induction of differentiation, irradiated BM-MSCs treated with exosomes exhibited reduced oil red staining and increased alizarin red s staining compared with those treated with radiation alone. These results demonstrated that BM-MSC-derived exosomes can restore the differentiation potential of irradiated BM-MSCs.

Wnt/beta-catenin signaling is a pivotal regulator of MSCs and plays an important role in adipogenic and osteogenic differentiation [52]. Activation of Wnt/β-catenin signaling inhibits BM-MSCs from undergoing adipogenesis and promotes osteogenesis [53, 54]. β-Catenin is the downstream of Wnt proteins, and recent studies have reported that exosomes and extracellular vesicles carry Wnt proteins to induce the activity of β-catenin on target cells [55, 56]. Zhang et al. [57] reported that human MSC-derived exosomes promote angiogenesis by transducing Wnt4 and activating Wnt/beta-catenin signaling in endothelial cells to repair deep second-degree burn skin injury. In our study, we found that after irradiation, exosome-treated BM-MSCs exhibited increased β-catenin expression compared with those treated with irradiation alone, as determined by RT-PCR, Western blot, and immunofluorescence. Consistently, we also found such differences in vivo. This finding indicates that BM-MSC-derived exosomes could activate Wnt/β-catenin signaling to restore the differentiation potential of irradiated BM-MSCs.

## Conclusions

We first identified that BM-MSC-derived exosomes alleviate radiation-induced bone loss in a rat model. The effects might be attributed to the functional recovery of recipient BM-MSCs, which is achieved by alleviating DNA and oxidative stress damage, rescuing proliferation inhibition, reducing cell senescence, and restoring the balance between adipogenic and osteogenic differentiation of irradiated BM-MSCs through Wnt/β-catenin signaling. Our findings suggest that BM-MSC-derived exosomes may be a promising cell-free therapeutic approach for the treatment of radiation-induced bone loss.

## Additional files

Additional file 1: **Figure S1.** Rat left tibiae were irradiated by using Co60 at a rate of 0.56 Gy/min. The remaining rat body parts were blocked by using lead bricks. (JPG 190 kb)

Additional file 2: **Figure S2.** Flow cytometry analyses of stem cell negative surface markers CD34 and CD45 (Santa Cruz, CA, USA) and positive markers CD29, CD44, and CD90 (Biolegend Inc., San Diego, USA). (A) P0 BM-MSCs were cultured for 3 or 5 days (stained with crystal violet) and subcultured to P1. (B) Alcian blue staining of chondrified micromass after chondrogenic induction for 21 days. Alizarin red S staining and Oil O staining of BM-MSCs after osteogenic or adipogenic induction for 14 days or 15 days. (JPG 1203 kb)

## Abbreviations

BMD: Bone mineral density
BM-MSCs: Bone marrow mesenchymal stem cells
BV/TV: Bone volume to total tissue volume ratio
Conn.D: Connective density
Ebf1: Early B cell factor 1
Ex: Exosomes
OPG: Osteoprotegerin
PPARγ: Peroxisome proliferator-activated receptor gamma
Rb: Retinoblastoma
Runx2: Runt-related transcription factor 2
SOD1: Superoxide dismutase 1
SOD2: Superoxide dismutase 2
Tb.N: Trabecular number
Tb.Th: Trabecular thickness
γH2AX: Phosphorylated histone H2A variant

## References

[1] Oronzo SD, Stucci S, Tucci M, Silvestris F (2015). Cancer treatment-induced bone loss (CTIBL): pathogenesis and clinical implications. Cancer Treat Rev.

[2] Wei RL, Jung BC, Manzano W, Sehgal V, Klempner SJ, Lee SP, Ramsinghani NS, Lall C (2016). Bone mineral density loss in thoracic and lumbar vertebrae following radiation for abdominal cancers. Radiother Oncol.

[3] Pacheco R, Stock H (2013). Effects of radiation on bone. Curr Osteoporos Rep.

[4] Baxter NN, Habermann EB, Tepper JE, Durham SB, Virnig BA (2005). Risk of pelvic fractures in older women following pelvic irradiation. JAMA.

[5] Schmeler KM, Jhingran A, Iyer RB, Sun CC, Eifel PJ, Soliman PT, Ramirez PT, Frumovitz M, Bodurka DC, Sood AK (2010). Pelvic fractures after radiotherapy for cervical cancer. Cancer-Am Cancer Soc.

[6] Wang Y, Zhu G, Wang J, Chen J (2016). Irradiation alters the differentiation potential of bone marrow mesenchymal stem cells. Mol Med Rep.

[7] Lo WJ, Lin CL, Chang YC, Bai LY, Lin CY, Liang JA, Li LY, Chao LM, Chiu CF, Chen CM, Yeh SP (2018). Total body irradiation tremendously impair the proliferation, differentiation and chromosomal integrity of bone marrow-derived mesenchymal stromal stem cells. Ann Hematol.

[8] Li J, Kwong DL, Chan GC (2007). The effects of various irradiation doses on the growth and differentiation of marrow-derived human mesenchymal stromal cells. Pediatr Transplant.

[9] Green DE, Adler BJ, Chan ME, Rubin CT (2012). Devastation of adult stem cell pools by irradiation precedes collapse of trabecular bone quality and quantity. J Bone Miner Res.

[10] Alessio N, Del GS, Capasso S, Di Bernardo G, Cappabianca S, Cipollaro M, Peluso G, Galderisi U (2015). Low dose radiation induced senescence of human mesenchymal stromal cells and impaired the autophagy process. Oncotarget.

[11] Greenberger JS, Epperly M (2009). Bone marrow–derived stem cells and radiation response. Semin Radiat Oncol.

[12] Zou Q, Hong W, Zhou Y, Ding Q, Wang J, Jin W, Gao J, Hua G, Xu X (2016). Bone marrow stem cell dysfunction in radiation-induced abscopal bone loss. J Orthop Surg Res.

[13] Zhang X, Xiang L, Ran Q, Liu Y, Xiang Y, Xiao Y, Chen L, Li F, Zhong JF, Li Z (2015). Crif1 promotes adipogenic differentiation of bone marrow mesenchymal stem cells after irradiation by modulating the PKA/CREB signaling pathway. Stem Cells.

[14] Hu L, Yin C, Zhao F, Ali A, Ma J, Qian A. Mesenchymal stem cells: cell fate decision to osteoblast or adipocyte and application in osteoporosis treatment. Int J Mol Sci. 2018;19. 10.3390/ijms19020360. 10.3390/ijms19020360 PMC5855582 29370110

[15] Wang C, Meng H, Wang X, Zhao C, Peng J, Wang Y (2016). Differentiation of bone marrow mesenchymal stem cells in osteoblasts and adipocytes and its role in treatment of osteoporosis. Med Sci Monit.

[16] Kuci Z, Bonig H, Kreyenberg H, Bunos M, Jauch A, Janssen JW, Skific M, Michel K, Eising B, Lucchini G, Bakhtiar S, Greil J, Lang P, Basu O, von Luettichau I, Schulz A, Sykora KW, Jarisch A, Soerensen J, Salzmann-Manrique E, Seifried E, Klingebiel T, Bader P, Kuci S (2016). Mesenchymal stromal cells from pooled mononuclear cells of multiple bone marrow donors as rescue therapy in pediatric severe steroid-refractory graft-versus-host disease: a multicenter survey. Haematologica.

[17] Gonzalez MA, Gonzalez-Rey E, Rico L, Buscher D, Delgado M (2009). Adipose-derived mesenchymal stem cells alleviate experimental colitis by inhibiting inflammatory and autoimmune responses. Gastroenterology.

[18] Hatzistergos KE, Quevedo H, Oskouei BN, Hu Q, Feigenbaum GS, Margitich IS, Mazhari R, Boyle AJ, Zambrano JP, Rodriguez JE, Dulce R, Pattany PM, Valdes D, Revilla C, Heldman AW, McNiece I, Hare JM (2010). Bone marrow mesenchymal stem cells stimulate cardiac stem cell proliferation and differentiation. Circ Res.

[19] Ma L, Aijima R, Hoshino Y, Yamaza H, Tomoda E, Tanaka Y, Sonoda S, Song G, Zhao W, Nonaka K, Shi S, Yamaza T (2015). Transplantation of mesenchymal stem cells ameliorates secondary osteoporosis through interleukin-17-impaired functions of recipient bone marrow mesenchymal stem cells in MRL/lpr mice. Stem Cell Res Ther.

[20] Kiernan J, Hu S, Grynpas MD, Davies JE, Stanford WL (2016). Systemic mesenchymal stromal cell transplantation prevents functional bone loss in a mouse model of age-related osteoporosis. Stem Cells Transl Med.

[21] Akbar MA, Lu Y, Elshikha AS, Chen MJ, Yuan Y, Whitley EM, Holliday LS, Chang LJ, Song S (2017). Transplantation of adipose tissue-derived mesenchymal stem cell (ATMSC) expressing alpha-1 antitrypsin reduces bone loss in ovariectomized osteoporosis mice. Hum Gene Ther.

[22] Liu S, Liu D, Chen C, Hamamura K, Moshaverinia A, Yang R, Liu Y, Jin Y, Shi S (2015). MSC transplantation improves osteopenia via epigenetic regulation of notch signaling in lupus. Cell Metab.

[23] Lai RC, Yeo RW, Lim SK (2015). Mesenchymal stem cell exosomes. Semin Cell Dev Biol.

[24] Phinney DG, Pittenger MF (2017). Concise review: MSC-derived exosomes for cell-free therapy. Stem Cells.

[25] He C, Zheng S, Luo Y, Wang B (2018). Exosome theranostics: biology and translational medicine. Theranostics.

[26] EL AS, Mager I, Breakefield XO, Wood MJ (2013). Extracellular vesicles: biology and emerging therapeutic opportunities. Nat Rev Drug Discov.

[27] Khan M, Nickoloff E, Abramova T, Johnson J, Verma SK, Krishnamurthy P, Mackie AR, Vaughan E, Garikipati VNS, Benedict C, Ramirez V, Lambers E, Ito A, Gao E, Misener S, Luongo T, Elrod J, Qin G, Houser SR, Koch WJ, Kishore R (2015). Embryonic stem cell-derived exosomes promote endogenous repair mechanisms and enhance cardiac function following myocardial infarction. Circ Res.

[28] Borrelli DA, Yankson K, Shukla N, Vilanilam G, Ticer T, Wolfram J (2018). Extracellular vesicle therapeutics for liver disease. J Control Release.

[29] Lin K, Yip H, Shao P, Wu S, Chen K, Chen Y, Yang C, Sun C, Kao G, Chen S, Chai H, Chang C, Chen C, Lee MS (2016). Combination of adipose-derived mesenchymal stem cells (ADMSC) and ADMSC-derived exosomes for protecting kidney from acute ischemia–reperfusion injury. Int J Cardiol.

[30] Murphy C, Withrow J, Hunter M, Liu Y, Tang YL, Fulzele S, Hamrick MW (2018). Emerging role of extracellular vesicles in musculoskeletal diseases. Mol Asp Med.

[31] Qi X, Zhang J, Yuan H, Xu Z, Li Q, Niu X, Hu B, Wang Y, Li X (2016). Exosomes secreted by human-induced pluripotent stem cell-derived mesenchymal stem cells repair critical-sized bone defects through enhanced angiogenesis and osteogenesis in osteoporotic rats. Int J Biol Sci.

[32] Zhao P, Xiao L, Peng J, Qian YQ, Huang CC (2018). Exosomes derived from bone marrow mesenchymal stem cells improve osteoporosis through promoting osteoblast proliferation via MAPK pathway. Eur Rev Med Pharmacol Sci.

[33] Yan Y, Jiang W, Tan Y, Zou S, Zhang H, Mao F, Gong A, Qian H, Xu W (2017). hucMSC exosome-derived GPX1 is required for the recovery of hepatic oxidant injury. Mol Ther.

[34] Zhou Y, Xu H, Xu W, Wang B, Wu H, Tao Y, Zhang B, Wang M, Mao F, Yan Y, Gao S, Gu H, Zhu W, Qian H (2013). Exosomes released by human umbilical cord mesenchymal stem cells protect against cisplatin-induced renal oxidative stress and apoptosis in vivo and in vitro. Stem Cell Res Ther.

[35] Antonyak MA, Cerione RA (2015). Emerging picture of the distinct traits and functions of microvesicles and exosomes. Proc Natl Acad Sci U S A.

[36] Kawai M, Rosen CJ (2010). PPARγ: a circadian transcription factor in adipogenesis and osteogenesis. Nat Rev Endocrinol.

[37] Jimenez MA, Akerblad P, Sigvardsson M, Rosen ED (2007). Critical role for Ebf1 and Ebf2 in the adipogenic transcriptional cascade. Mol Cell Biol.

[38] Ducy P, Zhang R, Geoffroy V, Ridall AL, Karsenty G (1997). Osf2/Cbfa1: a transcriptional activator of osteoblast differentiation. Cell.

[39] Martin TJ, Sims NA (2015). RANKL/OPG; critical role in bone physiology. Rev Endocr Metab Disord.

[40] Huang L, Ma W, Ma Y, Feng D, Chen H, Cai B (2015). Exosomes in mesenchymal stem cells, a new therapeutic strategy for cardiovascular diseases?. Int J Biol Sci.

[41] Lai RC, Chen TS, Lim SK (2011). Mesenchymal stem cell exosome: a novel stem cell-based therapy for cardiovascular disease. Regen Med.

[42] Lou G, Chen Z, Zheng M, Liu Y (2017). Mesenchymal stem cell-derived exosomes as a new therapeutic strategy for liver diseases. Exp Mol Med.

[43] Otero-Ortega L, Gomez DFM, Laso-Garcia F, Rodriguez-Frutos B, Medina-Gutierrez E, Lopez JA, Vazquez J, Diez-Tejedor E, Gutierrez-Fernandez M. Exosomes promote restoration after an experimental animal model of intracerebral hemorrhage. J Cereb Blood Flow Metab. 2018;38(5):767–779 10.1177/0271678X17708917 PMC5987932 28524762

[44] Liu X, Li Q, Niu X, Hu B, Chen S, Song W, Ding J, Zhang C, Wang Y. Exosomes secreted from human-induced pluripotent stem cell-derived mesenchymal stem cells prevent osteonecrosis of the femoral head by promoting angiogenesis. Int J Biol Sci. 2017;13:232–44. 10.7150/ijbs.16951 PMC5332877 28255275

[45] Hou J, Han Z, Jing Y, Yang X, Zhang S, Sun K, Hao C, Meng Y, Yu F, Liu X, Shi Y, Wu M, Zhang L, Wei L. Autophagy prevents irradiation injury and maintains stemness through decreasing ROS generation in mesenchymal stem cells. Cell Death Dis. 2013;4:e844. 10.1038/cddis.2013.338 PMC3824648 24113178

[46] Cmielova J, Havelek R, Soukup T, Jiroutová A, Visek B, Suchánek J, Vavrova J, Mokry J, Muthna D, Bruckova L, Filip S, English D, Rezacova M. Gamma radiation induces senescence in human adult mesenchymal stem cells from bone marrow and periodontal ligaments. Int J Radiat Biol. 2012;88:393–404. 10.3109/09553002.2012.666001 22348537

[47] Fekete N, Erle A, Amann EM, Fürst D, Rojewski MT, Langonné A, Sensebé L, Schrezenmeier H, Schmidtke-Schrezenmeier G (2015). Effect of high-dose irradiation on human bone-marrow-derived mesenchymal stromal cells. Tissue Eng Part C: Methods.

[48] Yu K, Kang K (2013). Aging-related genes in mesenchymal stem cells: a mini-review. Gerontology.

[49] Lunyak VV, Amaro-Ortiz A, Gaur M (2017). Mesenchymal stem cells secretory responses: senescence messaging secretome and immunomodulation perspective. Front Genet.

[50] Dudziak ME, Saadeh PB, Mehrara BJ, Steinbrech DS, Greenwald JA, Gittes GK, Longaker MT (2000). The effects of ionizing radiation on osteoblast-like cells in vitro. Plast Reconstr Surg.

[51] Szymczyk KH, Shapiro IM, Adams CS (2004). Ionizing radiation sensitizes bone cells to apoptosis. Bone.

[52] MacDonald BT, He X (2012). Frizzled and LRP5/6 receptors for Wnt/-catenin signaling. Csh Perspect Biol.

[53] Cawthorn WP, Bree AJ, Yao Y, Du B, Hemati N, Martinez-Santibañez G, MacDougald OA (2012). Wnt6, Wnt10a and Wnt10b inhibit adipogenesis and stimulate osteoblastogenesis through a β-catenin-dependent mechanism. Bone.

[54] Krishnan V (2006). Regulation of bone mass by Wnt signaling. J Clin Invest.

[55] Luga V, Zhang L, Viloria-Petit AM, Ogunjimi AA, Inanlou MR, Chiu E, Buchanan M, Hosein AN, Basik M, Wrana JL (2012). Exosomes mediate stromal mobilization of autocrine Wnt-PCP signaling in breast cancer cell migration. Cell.

[56] Menck K, Klemm F, Gross JC, Pukrop T, Wenzel D, Binder C (2013). Induction and transport of Wnt 5a during macrophage-induced malignant invasion is mediated by two types of extracellular vesicles. Oncotarget.

[57] Zhang B, Wu X, Zhang X, Sun Y, Yan Y, Shi H, Zhu Y, Wu L, Pan Z, Zhu W, Qian H, Xu W (2015). Human umbilical cord mesenchymal stem cell exosomes enhance angiogenesis through the Wnt4/beta-catenin pathway. Stem Cells Transl Med.

