# Correction to: BM-MSC-derived exosomes alleviate radiation-induced bone loss by restoring the function of recipient BM-MSCs and activating Wnt/β-catenin signaling

**DOI:** 10.1186/s13287-020-1553-x

**Published:** 2020-01-23

**Authors:** Rui Zuo, Minghan Liu, Yanqiu Wang, Jie Li, Wenkai Wang, Junlong Wu, Chao Sun, Bin Li, Ziwen Wang, Weiren Lan, Chao Zhang, Chunmeng Shi, Yue Zhou

**Affiliations:** 1 Department of Orthopedics, Xinqiao Hospital, Army Medical University (Third Military Medical University), Chongqing, 400038 People’s Republic of China; 2 Institute of Rocket Force Medicine, State Key Laboratory of Trauma, Burns and Combined Injury, Army Medical University (Third Military Medical University), Chongqing, 400038 People’s Republic of China

**Correction to: Stem Cell Res Ther**

**https://doi.org/10.1186/s13287-018-1121-9**

The original article [1] contains an error in Fig. 5 whereby sub-Fig. 5c, d & e are mistakenly mixed-up.

The correct version of Fig. 5 and the respective affected sub-figures can be viewed ahead and should be considered ahead of the incorrect Fig. 5 present in the original article.

**Fig. 5:**
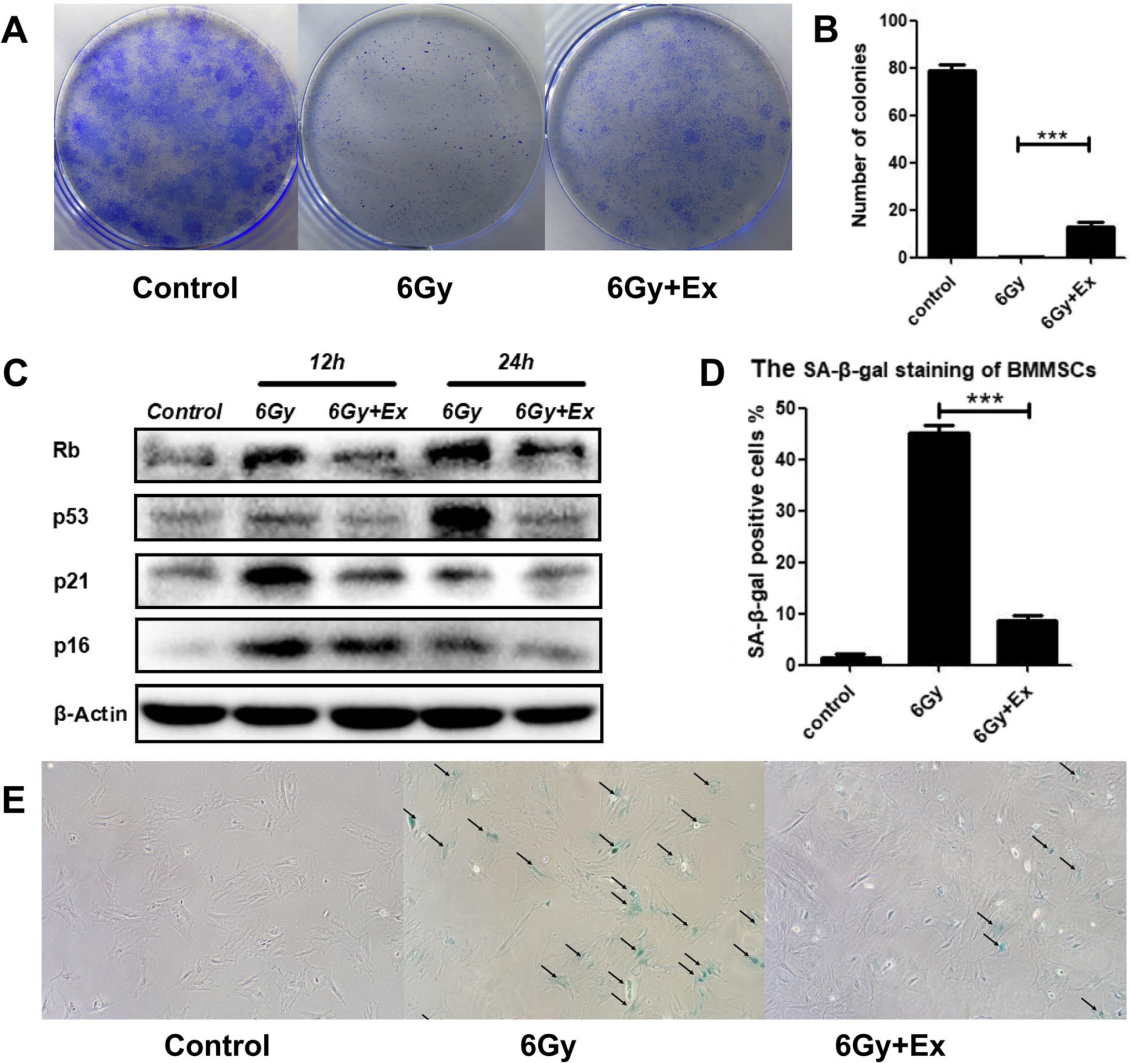
Exosomes rescued the inhibition of proliferation and decrease the senescence-associated protein expression of BM-MSCs after irradiation. **a** Colony-forming ability of BM-MSCs after irradiation. Colony formation was assessed by staining with crystal violet. **b** Quantitative analysis of colony-forming units. Data are presented as the mean ± SD (*n* = 3 independent experiments, *t* test). ***p < 0.001. **c** Western blot analysis of senescence-associated proteins, including Rb, P53, P21, and P16. **d** Percentage of SA-β-gal-positive cells under different treatments. Data are presented as the mean ± SD (*n* = 10 independent experiments, t test). ***p < 0.001. **e** Senescence-associated β-galactosidase (SA-β-gal) staining
