# Supplementary figures and images for: BM-MSC-derived exosomes alleviate radiation-induced bone loss by restoring the function of recipient BM-MSCs and activating Wnt/β-catenin signaling
Source: Stem Cell Res Ther. 2019 Jan 15;10:30. doi: 10.1186/s13287-018-1121-9 (PMC6334443; doi:10.1186/s13287-018-1121-9)

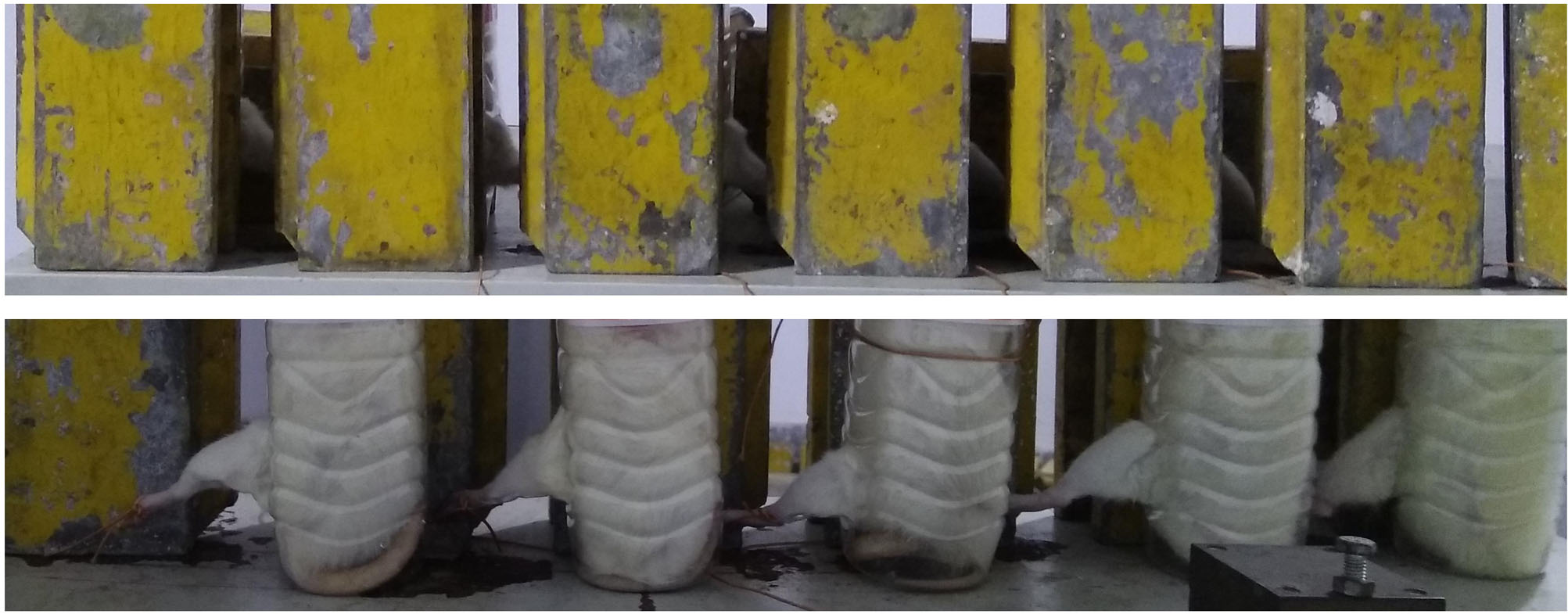

Supplement: Supplementary file 1 — Figure S1. Rat left tibiae were irradiated by using Co60 at a rate of 0.56 Gy/min. The remaining rat body parts were blocked by using lead bricks. (JPG 190 kb) [file 13287_2018_1121_MOESM1_ESM.jpg]

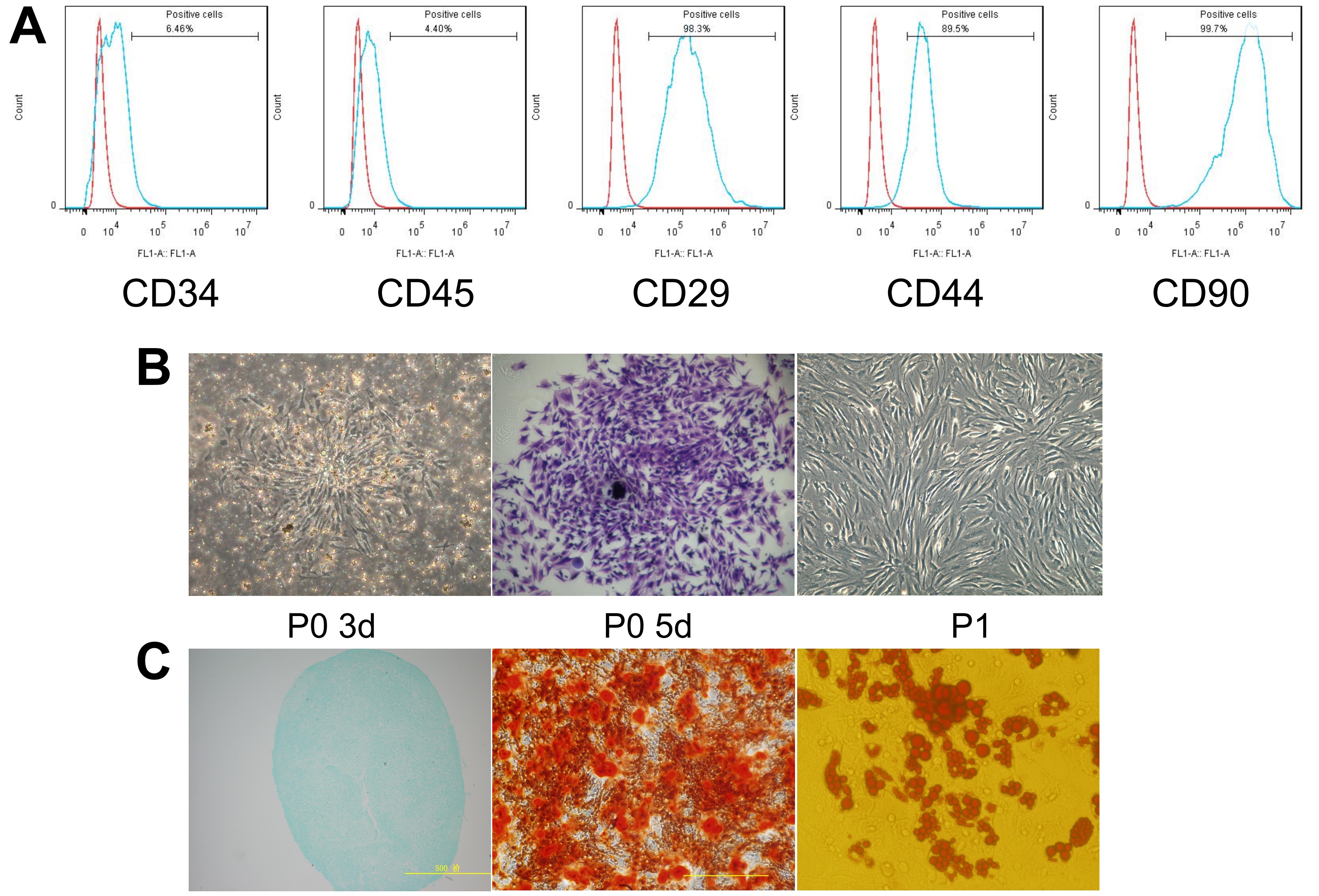

Supplement: Supplementary file 2 — Figure S2. Flow cytometry analyses of stem cell negative surface markers CD34 and CD45 (Santa Cruz, CA, USA) and positive markers CD29, CD44, and CD90 (Biolegend Inc., San Diego, USA). (A) P0 BM-MSCs were cultured for 3 or 5 days (stained with crystal violet) and subcultured to P1. (B) Alcian blue staining of chondrified micromass after chondrogenic induction for 21 days. Alizarin red S staining and Oil O staining of BM-MSCs after osteogenic or adipogenic induction for 14 days or 15 days. (JPG 1203 kb) [file 13287_2018_1121_MOESM2_ESM.jpg]
